# Association of parental body mass index (BMI) with child’s health behaviors and child’s BMI depend on child’s age

**DOI:** 10.1186/s40608-019-0232-x

**Published:** 2019-04-01

**Authors:** Che Young Lee, Tracey A. Ledoux, Craig A. Johnston, Guadalupe X. Ayala, Daniel P. O’Connor

**Affiliations:** 1 Department of Health and Human Performance, University of Houston, 3875 Holman Street Garrison Gym 104, Houston, TX 77204-6015 USA; 2 Division of Health Promotion and Behavioral Science, Graduate School of Public Health, San Diego State University, 5500 Campanile Drive, San Diego, 92182-4162 CA USA

**Keywords:** Childhood obesity, Body mass index, Physical activity, Nutrition, Path analysis

## Abstract

**Background:**

Parent’s and child’s body mass index (BMI) are strongly associated, but their relationship varies by child’s sex and age. Parental BMI reflects, among other factors, parents’ behaviors and home environment, which influence their child’s behaviors and weight. This study examined the indirect effect of parent’s BMI on child’s BMI via child health behaviors, conditional on child’s sex and age.

**Methods:**

Data from 2039 children and 1737 parents from eight cities of the U.S. involved in the Childhood Obesity Research Demonstration project tested the association between parental BMI and child’s percentage of 95th BMI percentile (%BMIp95). A generalized structural equation modeling approach to path analysis was used to estimate and test simultaneously the associations among parental BMI and child’s health behaviors and BMI across three age groups (preschool 2-4 yr., elementary 5-10 yr., and middle school 11-12 yr). Child’s health behaviors were examined as mediators.

**Results:**

Parental BMI was related to %BMIp95 across all age groups, and was strongest in 11-12 yr. children. Parental BMI was positively associated with boys’ fruit and vegetable (FV) intake and girls’ sugar-sweetened beverage (SSB) intake. Compared to 2-4 yr., older children had less FVs and physical activity, more screen time and SSB, and higher %BMIp95. Mediation effects were not significant.

**Conclusions:**

Parental BMI was associated with child’s %BMIp95 and some child behaviors, and this association was stronger in older children; older children also exhibited less healthy behaviors. Age- and sex-specific interventions that focus on age-related decreases in healthy behaviors and parental strategies for promoting healthy behaviors among at-risk children are needed to address this epidemic of childhood obesity.

## Background

About one third of children aged 2 to 19 years are classified as having overweight or obesity in the United States, and this high prevalence of childhood obesity has been present for decades [1]. Prevention of childhood obesity is a public health priority, because obesity in childhood increases risk of obesity in adulthood [2] and is associated with long-term adverse health consequences [3].

Numerous studies have reported a strong association between parent’s and child’s body mass index (BMI) [4, 5], and children whose parents had a healthy BMI exhibited healthier behaviors such as regular physical activity (PA) and improved dietary patterns [6], compared with children whose parents had higher BMI. Higher maternal BMI is related to higher child’s BMI and sedentary behavior [7], less fruit consumption [8], and more TV viewing [9, 10]. These results are consistent with the notion that parental BMI reflects parents’ health behaviors that influence their child’s health behaviors and ultimately weight status [11, 12]. Thus, the development of obesity in childhood and persistence into adulthood is not entirely explained by inheritable factors [13], but also by the health and parenting behaviors of parents/caregivers [4].

Although the sharing of genetic and behavioral factors between parents and children results in a similar propensity for obesity status [10, 14], the association of parent and child BMI has been shown to vary by child’s sex and age. Both son’s and daughter’s BMI has been reported to be significantly related to father’s BMI, while daughter’s BMI was significantly related to mother’s BMI only [15]. Two separate studies demonstrated that children’s PA was affected by shared environmental factors for parents and young children [16], but not for parents and adolescents [17]. This can be explained by a decreasing influence of parents on children’s behaviors as children mature and become more independent from their parents [14]. Moreover, older children’s behaviors and obesity status may be affected by school programs [18] and peer behaviors [19]. Given the influences of schools and peers on children’s health behaviors and consequently their BMI, we assume that the association of parental BMI on their child’s behaviors and BMI would be expected to vary as a function of child’s age. Thus, child age may moderate the association between parental BMI and child’s health behaviors and BMI.

This study investigated 1) the extent to which parental BMI was associated with the child’s health behaviors and BMI, 2) the role of child’s health behaviors as mediators between parental BMI and child’s BMI, and 3) whether these relationships are conditional on child’s sex and age. We hypothesized: 1) healthy parental BMI would be associated with healthier child behaviors and BMI, 2) healthy child behaviors would be related to a healthier child BMI, 3) the relation between parental BMI and child BMI would be partly mediated by the child’s health behaviors, and 4) these associations would vary by child’s sex and age.

## Methods

### Participants

This study was a secondary analysis of baseline data collected on the Childhood Obesity Research Demonstration project (CORD) [20]. CORD implemented integrated primary care and public health interventions across eight communities in three states in the U.S. to improve child and family health behaviors and to prevent and reduce childhood obesity among families eligible for benefits under Titles XIX (Medicaid) and XXI (Children’s Health Insurance Plan (CHIP)) of the Social Security Act, which are programs intended to serve families with low household income.

In 2012–2014, 2039 children aged 2–12 years and one of their parents or caregivers (*N* = 1737) were enrolled from eight communities in three states (Brawley, Calexico, and El Centro of California (CA); Fitchburg, Lowell, and New Bedford of Massachusetts (MA); and Austin and Houston of Texas (TX)). The TX project included only children with BMI ≥85th age and sex specific percentile, whereas the CA and MA projects also included healthy weight children. Demographic information for parents (sex, age, education, employment, and family income) and children (sex, age, and ethnicity) were collected from parents. Children were categorized into three age groups based on school status: preschool (2-4 yr), elementary (5-10 yr), and middle school (11-12 yr). The study was approved by the institutional review boards of the participating CORD organizations and institutions, and written informed consent of parents and assent of children were obtained prior to data collection.

### Anthropometric measures

Anthropometric measures were collected using the methods described in the National Health and Nutrition Examination Survey Anthropometry Procedures Manual [21]. Parent and child’s height and weight were recorded in centimeters (cm) to the nearest 0.1 cm and kilogram (kg) to the nearest 0.1 kg, respectively. Parental BMI was calculated using the Quetelet index equation (kg/m^2^). Each child’s relative BMI, computed as the percentage of the respective age and sex specific BMI 95th percentile value %BMIp95), was used as the child BMI variable, calculated using the CDC reference data and software algorithm [22]. This measure has been shown to be more appropriate for the heaviest children (i.e., those >97th percentile), a characteristic of this sample given the inclusion criteria in one site (TX) and the demographics of the other two sites (i.e., rural, racially/ethnically diverse, low-income [23]). In addition, it has better statistical properties for comparisons than other BMI-derived measures for children [24].

### Child health behavior variables

Child health behaviors were measured by surveying parents using a standard set of items selected from previously validated instruments [20, 25]. Child’s times per day eating fruits and vegetables (FVs) (sum of 2 items) and sugar-sweetened beverages (SSBs) (sum of 2 items) were assessed using items from the School Physical Activity and Nutrition project survey [26] and the Child and Adolescent Trial for Cardiovascular Health (CATCH) Kids Club After-school questionnaire [27]. Number of days per week engaged in 60 min or more of PA was assessed using one item from the Youth Risk Behavior Survey [28]. The parent selected from 0 to 7 days for their child, which resulted in a highly negatively skewed distribution, so the observed responses were dichotomized into 7 days/week (every day) versus less than 7 days/week (not every day) for analyses. Total hours and minutes per week of screen time (TV/DVD, computer/video game, etc.) were computed from hours and minutes per weekday and weekend day for screen time collected using four items from the CATCH Kids Club After-school questionnaire [27].

### Analyses

**Fig. 1:**
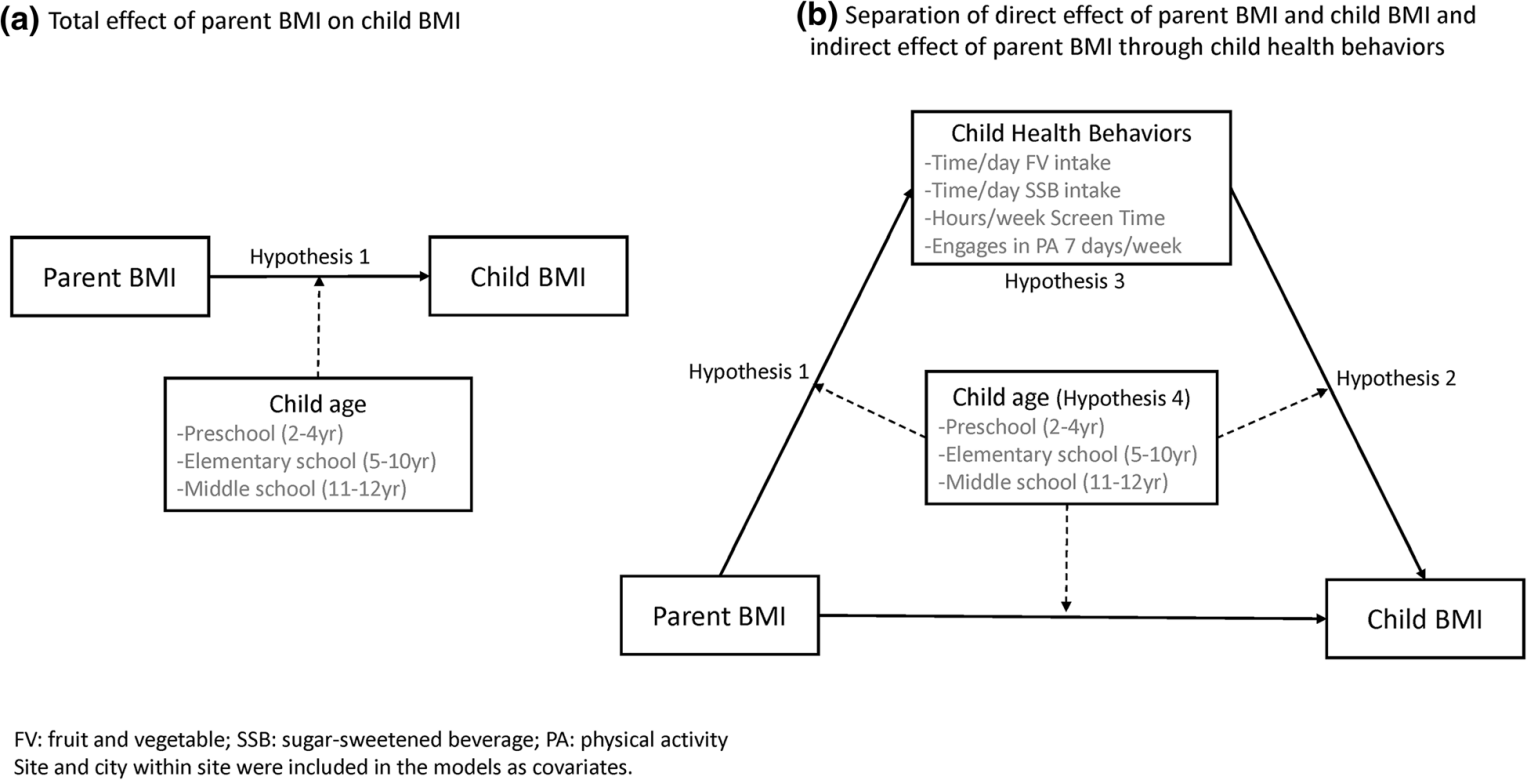
Analytical models tested in the study. (**a**) Total effect of parent BMI on child BMI. (**b**) Direct effect of parent and child BMI and indirect effect through child health behaviors. FV: fruit and vegetable; SSB: sugar-sweetened beverage; PA: physical activity. Site and city within site were included in the models as covariates

Separate analyses were conducted for boys and girls. Descriptive statistics were reported as means and standard deviations (mean ± SD) or percentages (%). A generalized structural equation modeling approach to path analysis was used to estimate and test simultaneously the associations between parental BMI and child’s BMI, parental BMI and child’s health behaviors, and child’s health behaviors and BMI among the three child age groups (Fig. 1). Associations were modeled using linear links and normal distributions, except for children’s PA, which was a dichotomous variable and was modeled using a log link and binomial distribution. Parental BMI was mean-centered so that interaction effects could be interpreted at the average BMI of the parents. State (CA, MA, and TX) and city within state (Brawley, Calexico, El Centro, Fitchburg, Lowell, New Bedford, Austin, and Houston) were included as covariates in all statistical models to adjust for mean differences among study sites.

Four separate analyses were conducted to test sequentially: 1) direct effects of parental BMI on the child’s %BMIp95 (Hypothesis 1) and whether the direct effects varied by age group (Hypothesis 4, Fig. 1a); 2) associations in the full path model (Fig. 1b) between parental BMI and the child’s health behaviors (Hypothesis 1), and between child’s health behaviors and %BMIp95 (Hypothesis 2) and whether these effects varied by age group (Hypothesis 4, Fig. 1b), and 3) indirect (mediation) effects (Hypothesis 3), the path from parental BMI through the child’s health behavior to the child’s %BMIp95 (Fig. 1b), and whether the mediation effect varied by age. All analyses were adjusted for site and for different sample sizes among the age groups and across cities. All statistical analyses were performed using Stata 14.2 (Stata Corp, Texas, USA), and significance was defined as *p* < .05.

## Results

Only 4% of adults reported being non-parent guardians, and 91.5% reported being the mother of the enrolled child (Table 1). About 64% of parents had graduated high school, less than 50% were employed, and about 70% were living in households below the federal poverty level (FPL). Overall parental BMI was 31.6 ± 7.4.

**Table 1:** Characteristics of parents across the three sites

Parent		MA	CA	TX	Total
N		421	804	512	1737
Gender	Female (%)	90.7%	98.4%	95.9%	95.0%
Age (yr)	Mean (SD)	33.4 ± 8.2	36.2 ± 8.4	34.4 ± 7.0	34.6 ± 7.9
Relation with child	Mother (%)	86.7%	93.7%	94.1%	91.5%
Education	High school graduate (%)	68.8%	68.7%	55.6%	64.4%
Employment	Work for pay (%)	52.0%	39.7%	47.5%	46.4%
Income	Less than $15,000 (%)	53.2%	43.5%	60.0%	51.9%
Poverty Index	% below FPL	73.8%	60.6%	72.4%	68.9%
BMI		29.8 ± 7.3	31.8 ± 7.2	33.2 ± 7.8	31.6 ± 7.4

Mean ± SD

*MA*: Massachusetts; *CA*: California; *TX*: Texas

*FPL*: federal poverty level

*BMI*: body mass index, kg/m^2^

*Age*: years

The total number of boys and girls were 1014 and 1025, respectively (Table 2). More than 80% of children were reported to be Hispanic. Due to the inclusion criterion in TX for children to be ≥85th BMI percentile, the TX children had a higher mean %BMIp95. The TX children also had less frequent FV intake, more frequent SSB intake, more hours of screen time, and a lower proportion participating every day in ≥60 min/day of PA, compared to the MA and CA children. The percentage of children with normal weight, overweight, and obesity were 22.3, 21.8, and 55.9%, respectively.

**Table 2:** Characteristics of children across the three sites

Child		MA	CA	TX	Total/Average
Boys	N	218	552	244	1014
	Age (yr)	6.6 ± 3.3	6.9 ± 2.7	7.7 ± 2.8	7.1 ± 2.9
	Race (% of Hispanic)	57.8%	98.0%	88.9%	81.6%
	% of 95th BMI percentile	95.1 ± 18.9	98.8 ± 19.7	117.6 ± 19.2	103.9 ± 19.3
	FV Intake (times/day)	2.3 ± 1.7	2.3 ± 1.5	1.9 ± 1.3	2.2 ± 1.5
	SSB Intake (times/day)	0.7 ± 0.9	1.1 ± 1.2	1.3 ± 1.2	1.0 ± 1.1
	Screen Time (hrs/wk)	23.2 ± 15.8	27.2 ± 16.1	28.5 ± 16.6	26.3 ± 16.2
	7 days/wk. of PA 60 min/day	76.2%	43.5%	19.7%	46.4%
Girls	N	203	567	255	1025
	Age (yr)	6.5 ± 3.1	6.9 ± 2.6	7.7 ± 2.7	7.0 ± 2.8
	Race (% of Hispanic)	69.0%	98.9%	85.9%	84.6%
	% of 95th BMI percentile	92.1 ± 19.8	96.2 ± 20.3	115.99 ± 16.8	101.4 ± 18.6
	FV Intake (times/day)	2.3 ± 1.4	2.4 ± 1.5	2.3 ± 1.4	2.3 ± 1.5
	SSB Intake (times/day)	0.8 ± 1.1	1.0 ± 1.0	1.2 ± 1.3	1.0 ± 1.1
	Screen Time (hrs/wk)	21.7 ± 13.0	22.3 ± 12.8	26.5 ± 15.5	24.2 ± 13.8
	7 days/wk. of PA 60 min/day	69.5%	42.3%	14.9%	42.2%
Overall	N	421	1119	499	2039
	Age (yr)	6.5 ± 3.2	6.9 ± 2.7	7.7 ± 2.8	7.1 ± 2.9
	Race (% of Hispanic)	63.4%	98.5%	87.9%	83.1%
	% of 95th BMI percentile	93.6 ± 19.4	97.5 ± 20.0	116.80 ± 18.0	102.7 ± 19.0
	FV Intake (times/day)	2.3 ± 1.6	2.4 ± 1.5	2.1 ± 1.4	2.3 ± 1.5
	SSB Intake (times/day)	0.8 ± 1.0	1.1 ± 1.1	1.3 ± 1.3	1.0 ± 1.1
	Screen Time (hrs/wk)	22.5 ± 14.4	24.8 ± 14.5	27.5 ± 16.1	25.3 ± 15.0
	7 days/wk. of PA 60 min/day	72.9%	42.9%	17.3%	44.3%

Mean ± SD

*MA*: Massachusetts, *CA*: California, *TX*: Texas

FV intake: time(s) per day fruit and vegetable intake; SSB intake: time(s) per day sugar-sweetened beverage (i.e., soda, punch) intake; screen time: total hour(s) of screen time (i.e., computer, TV, video game) during 5 weekdays and 2 weekend days; 7 days/wk. of PA 60 min/day: percentage of children who every day do physical activity that make the child breath hard for 60 min or more

Age group differences for child’s BMI and health behaviors after adjusting for parental BMI and city were reported in Table 3. Compared to the preschool boys, elementary school boys had significantly higher %BMIp95. Elementary and middle school boys showed significantly higher screen time (*p* < .001) and lower FV intake (*p* < .001) and PA (elementary: *p* = .001; middle school: *p* < .001) than preschool boys. Elementary and middle school girls showed higher screen time (*p* < .001) and lower FV intake and PA (elementary: p < .001; middle school: p = .001) than preschool girls. SSB intake in middle school girls were significantly higher (*p* = .023) than preschool girls.

**Table 3:** Age differences of child’s BMI and health behaviors in both boys and girls

Child		Preschool	Elementary school	Middle School	Total/Average
Boys	N	299	617	98	1014
	% of 95th BMI percentile	99.9 ± 1.1	103.3 ± 0.8*	100.1 ± 1.9	101.1 ± 1.3
	FV Intake (times/day)	2.6 ± 0.1	1.9 ± 0.1***	1.9 ± 0.2***	2.1 ± 0.1
	SSB Intake (times/day)	0.9 ± 0.1	1.1 ± 0.1	1.1 ± 1.1	1.0 ± 0.4
	Screen Time (hrs/wk)	22.6 ± 0.1	27.9 ± 0.7***	30.5 ± 1.6***	27.0 ± 1.1
	7 days/wk. of PA 60 min/day (OR)	0.6 ± 0.0	0.5 ± 0.0**	0.4 ± 0.1***	0.5 ± 0.0
Girls	N	282	666	77	1025
	% of 95th BMI percentile	99.1 ± 1.2	100.8 ± 0.9	98.2 ± 2.1	99.3 ± 1.4
	FV Intake (times/day)	2.6 ± 0.1	2.2 ± 0.1***	2.0 ± 0.2**	2.3 ± 0.1
	SSB Intake (times/day)	0.9 ± 0.1	1.0 ± 0.1	1.2 ± 0.1*	1.1 ± 0.1
	Screen Time (hrs/wk)	20.6 ± 0.8	24.5 ± 0.6***	29.2 ± 1.5***	24.8 ± 1.0
	7 days/wk. of PA 60 min/day (OR)	0.6 ± 0.0	0.4 ± 0.0***	0.3 ± 0.1**	0.4 ± 0.0

Values are model-predicted means±SE, adjusted for mean of parental BMI and different numbers of children across the sites and cities

*p < .05, **p = .001, ***p < .001

Preschool is the reference group

### Direct effect of parental BMI on child’s BMI among age groups

Parental BMI was significantly positively associated with child’s %BMIp95 in both boys and girls (Hypothesis 1, *p* < .001). The association of parental BMI and child’s %BMIp95 increased significantly with child’s age (i.e., Hypothesis 4, parent BMI*child age interaction) among boys (*p* = .016) and girls (*p* = .019). A positive one-unit difference in parental BMI was associated with a 0.3, 0.8, and 0.7% higher %BMIp95 in preschool, elementary, and middle school boys, respectively. A positive one-unit difference in parental BMI was associated with a 0.5, 0.9, and 1.1% higher %BMIp95 in preschool, elementary, and middle school girls, respectively.

### Association between parental BMI and child’s health behaviors

Averaged across all age groups, parental BMI was significantly positively associated with FV intake (0.2 more times per day per 10-unit difference in parent BMI, *p* = .029) in boys and SSB intake (0.2 more times per day per 10-unit difference in parent BMI, *p* = .007) in girls (Hypothesis 1). However, parental BMI was not associated with SSB consumption (*p* = .931), screen time (*p* = .833), or PA (*p* = .515) in boys and FV intake (*p* = .815), screen time (*p* = .379), or PA (*p* = .794) in girls (Hypothesis 1, data not shown).

In comparing the age groups, higher parental BMI was associated with more screen time (*p* = .045) and engaging in PA seven days/week (*p* = .031) in elementary school boys and more FV intake (*p* = .013) in middle school boys. Age did not moderate the association between parental BMI and girls’ health behaviors (Hypothesis 4, Tables 4 and 5).

**Table 4:** The associations among parental BMI, child’s health behaviors, and child’s BMI in boys

Boys	P-BMI ➔ FV	P-BMI ➔ SSB	P-BMI ➔ Screen Time	P-BMI ➔ PA
Constant	2.51 ± 0.15***	1.05 ± 0.11***	22.99 ± 1.62***	0.53 ± 0.12*
Age2	−0.68 ± 0.10***	0.11 ± 0.08	5.00 ± 1.13***	0.57 ± 0.09***
Age3	−0.84 ± 0.17***	0.14 ± 0.13	7.95 ± 1.90***	0.34 ± 0.10***
P-BMI	0.001 ± 0.01	0.01 ± 0.008	−0.06 ± 0.12	0.97 ± 0.02*
P-BMI*Age2	−0.003 ± 0.01	−0.001 ± 0.01	0.25 ± 0.15*	1.04 ± 0.02*
P-BMI*Age3	0.06 ± 0.03*	−0.03 ± 0.02	−0.12 ± 0.28	1.03 ± 0.04
	FV ➔ C-BMI	SSB ➔ C-BMI	Screen Time ➔ C-BMI	PA ➔ C-BMI
Constant	112.54 ± 2.68***	114.49 ± 2.15***	113.63 ± 2.46***	114.17 ± 2.20***
Age2	4.17 ± 2.59	0.80 ± 1.78	− 0.69 ± 2.42	3.92 ± 1.90*
Age3	1.11 ± 3.92	1.60 ± 3.05	−3.59 ± 4.20	1.39 ± 2.86
P-BMI	0.25 ± 0.73*	0.26 ± 0.14*	0.26 ± 0.14*	0.24 ± 0.14*
P-BMI*Age2	0.55 ± 0.17**	0.53 ± 0.17**	0.52 ± 0.17**	0.57 ± 0.17**
P-BMI*Age3	0.46 ± 0.34	0.41 ± 0.33	0.47 ± 0.33	0.45 ± 0.33
Behavior	0.46 ± 0.73	−0.75 ± 0.96	0.004 ± 0.068	−14.05 ± 4.80*
Behavior*Age2	−0.48 ± 0.90	1.97 ± 1.16*	0.13 ± 0.08	3.33 ± 2.66
Behavior*Age3	−0.53 ± 1.65	−1.54 ± 1.93	0.11 ± 0.13	6.96 ± 4.50

All values are fixed regression coefficient ± SE, except values of P-BMI ➔ PA are odd ratios (OR) ± SE

*p < .05, **p = .001, ***p < .001

Constant: preschool (reference groups); Age2: elementary; Age3: middle school

P-BMI: mean-centered parental BMI; C-BMI: child percent of 95th BMI percentile (%BMIp95)

Behaviors: FV (fruit and vegetable intake [times per day]), SSB (sugar-sweetened beverage intake [times per day]), screen time (TV, DVD, computer, video game [hours per week]), and PA (physical activity [7 days/week vs. < 7 days/week]) of child

**Table 5:** The associations among parental BMI, child’s health behaviors, and child’s BMI in girls

Girls	P-BMI ➔ FV	P-BMI ➔ SSB	P-BMI ➔ Screen Time	P-BMI ➔ PA
Constant	2.52 ± 0.15***	0.92 ± 0.12***	19.68 ± 1.40***	0.37 ± 0.10***
Age2	−0.38 ± 0.10***	0.12 ± 0.08	3.78 ± 0.94***	0.52 ± 0.08***
Age3	−0.67 ± 0.19***	0.30 ± 0.14*	8.36 ± 1.73***	0.43 ± 0.13*
P-BMI	−0.008 ± 0.01	0.005 ± 0.009	−0.067 ± 0.105	1.02 ± 0.02
P-BMI*Age2	0.005 ± 0.04	0.015 ± 0.01	0.15 ± 0.13	0.98 ± 0.02
P-BMI*Age3	0.026 ± 0.02	0.021 ± 0.02	0.26 ± 0.22	0.95 ± 0.04
	FV ➔ C-BMI	SSB ➔ C-BMI	Screen Time ➔ C-BMI	PA ➔ C-BMI
Constant	113.86 ± 2.76***	112.78 ± 2.17***	114.17 ± 2.60***	112.22 ± 2.22***
Age2	2.12 ± 2.61	1.53 ± 1.76	−0.70 ± 2.54	2.54 ± 1.85
Age3	1.96 ± 4.40	−1.71 ± 3.35	−8.12 ± 4.78*	1.89 ± 3.12
PBMI	0.48 ± 0.15**	0.49 ± 0.15**	0.48 ± 0.15**	0.51 ± 0.15**
P-BMI*Age2	0.37 ± 0.18*	0.37 ± 0.18*	0.37 ± 0.18*	0.35 ± 0.18*
P-BMI*Age3	0.65 ± 0.31*	0.60 ± 0.31*	0.55 ± 0.31*	0.46 ± 0.31
Behavior	−0.56 ± 0.75	−0.33 ± 1.03	−0.09 ± 0.09	−15.83 ± 5.30*
Behavior*Age2	−0.45 ± 0.89	−0.16 ± 1.22	0.10 ± 0.10	3.67 ± 2.65
Behavior*Age3	−1.98 ± 1.76	0.23 ± 2.02	0.26 ± 0.16	10.39 ± 5.07*

All values are fixed regression coefficient ± SE, except values of P BMI ➔ PA are odd ratios (OR), ±SE

*p < .05, **p = .001, ***p < .001

Constant: preschool (reference groups); Age2: elementary; Age3: middle school

PBMI: mean-centered parental BMI; CBMI: child percent of 95th BMI percentile (%BMIp95)

Behaviors: FV (fruit and vegetable intake [times per day]), SSB (sugar-sweetened beverage intake [times per day]), screen time (TV, DVD, computer, video game [hours per week]), and PA (physical activity [7 days/week vs. < 7 days/week]) of child

### Association between child’s health behaviors and child’s BMI

Across all age groups, FV intake (*p* = .262, *p* = .278), SSB intake (*p* = .227, *p* = .372), and screen time (*p* = .480, *p* = .258) were not significantly related to boys’ and girls’ %BMIp95, but engaging in PA seven days/week (*p* = .002) was significantly associated with healthier %BMIp95 among boys and girls. Middle school girls who did not engage in PA seven days/week (*p* = .020) and elementary school boys who consumed SSB more times/day (*p* = .046) had significantly higher %BMIp95.

Preschool boys ate FVs 0.7 and 0.8 more times/day than elementary and middle school boys, respectively (*p* < .001). SSB intake did not differ significantly across boys’ age groups. Compared to preschool boys, elementary and middle school boys had significantly longer screen time (5 h and 8 h, respectively, *p* < .001), and were less likely to engage in PA seven days/week (*p* < .001, elementary: OR = 0.57; middle school: OR = 0.34) (Table 4).

Preschool girls ate FVs 0.4 and 0.7 more times/day than elementary and middle school girls, respectively (*p* < .001). Middle school girls consumed SSB 0.3 more times/day than preschool girls (*p* = .018). Compared to preschool girls, elementary and middle school girls had significantly longer screen time (3.8 h and 8.4 h, respectively, *p* < .001), and were less likely to engage in PA seven days/week (elementary: OR = 0.52, *p* < .001; middle school: OR = 0.43, *p* = .003) (Table 5).

### Indirect (mediation) effects

In both boys and girls, across and within all age groups, none of the indirect effects of parental BMI on child’s %BMIp95 via child’s health behaviors were statistically significant (Hypothesis 3). Thus, there are no mediatory effects of child’s health behaviors on the relationship observed between parental BMI and child’s %BMIp95.

## Discussion

In this study, parental BMI was positively related to child’s %BMIp95 in both boys and girls, as previous studies have reported [4, 5]. The 90% of enrolled adults being mothers may have resulted in the stronger observed association of mothers’ BMI on %BMIp95 in daughters since the association of BMI in mother-daughter dyads is higher than in mother-son, father-daughter, or father-son dyads [5]. In our data, older boys (elementary) and girls (elementary and middle school) showed a stronger positive association between %BMIp95 and parental BMI, compared to the preschool-aged children. Previous studies found that obesity status in older children was affected by both inheritable traits from parents and shared environment over time and emphasized that environmental effects were important determinants to develop behavior patterns and obesity among adolescents [13, 29]. One potential explanation for our results is that factors common to both parents and children, who live in the same household, including genetic, environmental, and sociocultural influences, may result in higher %BMIp95 in older children and increase the association with parental BMI.

An assumption in interpreting our results is that parental BMI is an indicator of genetic, environmental, and sociocultural factors common to both parents and children, and potentially long-term parental dietary, PA, and sedentary behaviors, and that those health behaviors would influence their child’s health behaviors and BMI [11, 12]. Thus, we expected that unhealthy parental BMI would be associated with child’s unhealthy behaviors such as less FV and more SSB intake (Hypothesis 1). Consistent with energy balance theory, we hypothesized that unhealthy child behaviors related to energy intake (FV and SSB intake) and to energy expenditure (PA and screen time) would be related to their %BMIp95 (Hypothesis 2). In the present study, a large difference in parental BMI was positively associated with FV intake in boys and SSB intake in girls (although the effect size is very small, it was statistically significant due to the large sample size), but not with child’s screen time and PA. Previous studies found an association of higher parental BMI with their children viewing more TV [9, 10] and engaging in less PA [6]. Additionally, only children’s PA was associated with their %BMIp95 in this study, whereas other studies found relationships between children’s BMI and their dietary and sedentary behaviors [6, 30, 31].

One explanation for inconsistencies between our results and previous studies may be due to different measures of child’s health behaviors. In our study, children’s FV and SSB intake were measured as times/day of the prior day, which do not provide a complete quantification of a child’s dietary intake, whereas previous studies measured both frequency and portion sizes [32]. Nevertheless, our data indicate that parental BMI is a correlate of some child’s health behaviors and %BMIp95, and the survey questions that we used in this study have been validated and used in previous studies [33].

The current study observed age differences in the associations among parental BMI, child’s health behaviors, and %BMIp95 (Hypothesis 4). Overall, preschool-aged children showed healthier behaviors such as more frequent FV intake, less frequent SSB intake, less weekly screen time, and higher proportion engaging in daily PA compared to elementary and middle school children in both boys and girls. In particular, engaging in PA seven days/week was lower, while screen time was higher, among the older children than the youngest children. These results are consistent with reports that suggest a decrease of PA is significantly associated with an increase of screen time among children and adolescents [10, 34, 35]. The reasons why child’s PA declines with age are unclear, but it is possible that social support factors (parental influence, schools’ academic programming and facilities, peers’ activity, etc.) may be associated with decreases in opportunities for moderate to vigorous physical activity (MVPA) among adolescents [36, 37]. In this study, there was significant association of higher parental BMI and engagement in PA every day in elementary school boys. One potential explanation of this result could be that boys’ PA are less dependent on their mother’s PA and BMI, since boys’ PA were more associated with their fathers [38]. Higher SSB intake and screen time in the oldest girls were observed in this study, consistent with research that reported an association between higher soda intake and longer TV viewing time in older children [39]. Higher FV intake in middle school boys with higher parental BMI could be explained by greater overall frequency of food consumption, including more fruit and vegetables, with larger parent body size [40]. Another potential explanation is that the parents with higher BMI may be more concerned with obesity in their sons and provide a better diet as reflected by their higher FV consumption.

Finally, we expected child’s health behaviors to be mediators partly explaining the association between parent’s and child’s BMI (Hypothesis 3). However, none of the mediation effects were statistically significant. This was due to either non-significant associations between parental BMI and child’s health behaviors or between child’s health behaviors and %BMIp95, or both. Our model showed that some of the child’s health behaviors (e.g., FV and SSB intake, and PA) were associated with parental and child’s BMI, but the direct relationship between parental BMI and child’s BMI remained relatively unchanged. One previous study found a stronger relationship between parent’s and child’s health behaviors compared to the relationship between parental and child’s BMI [39]. Although parental health behaviors were not assessed across all sites, parents’ behavioral influence on modifiable child’s health behaviors may affect child’s BMI and thus explain part of the association between parental and child’s BMI. Such behaviors may be opportunities to consider when designing interventions with a goal of changing behaviors in both parent and child to affect BMI. Caution is warranted in interpreting that a direct or indirect association indicates that “blame” should be placed on individuals, such as viewing parents as “the” causal agent of obesity in childhood. Our data do not suggest this. Instead, these associations should be viewed as opportunities to determine factors that may impact obesity in children. Because obesity is an intractable disease with multiple etiologies, “blaming” individuals (either parents or children) is counterproductive and fails to consider the environmental, genetic, epigenetic, and biological aspects of obesity.

This study has a number of limitations. First, the sample was primarily Hispanic families who were eligible for Medicaid and CHIP benefits, so the results may not generalize to populations with a different ethnicity or higher household income. Second, the cross-sectional data allows for only evaluating associations among parental BMI and child’s health behaviors and %BMIp95; there may be unmeasured causal variables and paths that were not included in the analyses. Third, the survey items did not reflect long-term child health behaviors, asking only about behaviors on a single day or week, and self-reported behaviors may not be as accurate as more objective measures. Fourth, parental behavior data were not consistently collected across the sites and were therefore not available for our analyses; parent behaviors may be more directly and strongly related to child health behaviors than is parent BMI. Fifth, the oldest age group sample sizes were smaller than the other age groups, thus limiting the precision of the estimates for that group.

Despite these limitations, this study included low-income families from different states and cities across the USA, which allows broader generalization of the results compared to single-site studies. Investigation of age differences of the relationships among parental BMI, child’s health behaviors, and child’s BMI is a novel aspect. Age-specific associations may be informative for considering different intervention strategies, such as providing interventions for the family and home environment for preschool children, but including additional interventions for older children, since older children spend much time at school as well as home, make decisions more independently, and are influenced by peer groups in addition to parents [14, 19].

## Conclusion

This study demonstrated a large association between parent BMI and child’s %BMIp95 but failed to detect any mediation through child health behaviors. The association between parental BMI and older children’s %BMIp95 was stronger compared to younger children. Older children also had unhealthier behaviors such as less daily FV intake and PA engagement and more weekly screen time and SSB intake; these unhealthy behaviors were associated with their higher %BMIp95. Parental BMI would impact unhealthy behaviors and obesity in their children, but our results are consistent with the notion that childhood obesity may be affected by multi-factors such as environmental factors, inheritable factors, parental behaviors, and a child’s own unhealthy behaviors. Thus, interventions for the prevention and control of childhood obesity may consider focusing on simultaneously changing the health behaviors of both parents and children. Our findings are also consistent with the notion that early life (before age 5) may be the best opportunity for interventions to prevent childhood obesity, before children develop their own unhealthy behaviors and weight status.

## Abbreviations

%BMIp95: the percentage of 95th BMI percentile
BMI: body mass index
CA: California
CATCH: Child and Adolescent Trial for Cardiovascular Health
CORD: Childhood Obesity Research Demonstration
FV: fruit and vegetables
MA: Massachusetts
PA: physical activity
SSB: sugar-sweetened beverage
TX: Texas

## References

[1] Ogden CL, Carroll MD, Kit BK, Flegal KM (2014). Prevalence of childhood and adult obesity in the United States, 2011-2012. Jama.

[2] Singh AS, Mulder C, Twisk JW, van Mechelen W, Chinapaw MJ (2008). Tracking of childhood overweight into adulthood: a systematic review of the literature. Obes Rev.

[3] Biro FM, Wien M (2010). Childhood obesity and adult morbidities. Am J Clin Nutr.

[4] Farajian P, Panagiotakos DB, Risvas G, Malisova O, Zampelas A (2014). Hierarchical analysis of dietary, lifestyle and family environment risk factors for childhood obesity: the GRECO study. Eur J Clin Nutr.

[5] Liu Y, Chen HJ, Liang L, Wang Y (2013). Parent-child resemblance in weight status and its correlates in the United States. PLoS One.

[6] Kosti RI, Panagiotakos DB, Tountas Y, Mihas CC, Alevizos A, Mariolis T, Papathanassiou M, Zampelas A, Mariolis A (2008). Parental body mass index in association with the prevalence of overweight/obesity among adolescents in Greece; dietary and lifestyle habits in the context of the family environment: the Vyronas study. Appetite.

[7] Sijtsma A, Sauer PJ, Corpeleijn E (2015). Parental correlations of physical activity and body mass index in young children--he GECKO Drenthe cohort. Int J Behav Nutr Phys Act.

[8] Morello MI, Madanat H, Crespo NC, Lemus H, Elder J (2012). Associations among parent acculturation, child BMI, and child fruit and vegetable consumption in a Hispanic sample. J Immigr Minor Health.

[9] Maffeis C, Talamini G, Tato L (1998). Influence of diet, physical activity and parents' obesity on children's adiposity: a four-year longitudinal study. Int J Obes Relat Metab Disord.

[10] Steffen LM, Dai S, Fulton JE, Labarthe DR (2009). Overweight in children and adolescents associated with TV viewing and parental weight: project HeartBeat. Am J Prev Med.

[11] Ventura AK, Birch LL (2008). Does parenting affect children's eating and weight status?. Int J Behav Nutr Phys Act.

[12] Carriere G (2003). Parent and child factors associated with youth obesity. Health Rep.

[13] Nelson MC, Gordon-Larsen P, North KE, Adair LS (2006). Body mass index gain, fast food, and physical activity: effects of shared environments over time. Obesity (Silver Spring).

[14] Silventoinen K, Rokholm B, Kaprio J, Sorensen TI (2010). The genetic and environmental influences on childhood obesity: a systematic review of twin and adoption studies. Int J Obes.

[15] Shafaghi K, Shariff ZM, Taib MN, Rahman HA, Mobarhan MG, Jabbari H (2014). Parental body mass index is associated with adolescent overweight and obesity in Mashhad. Iran Asia Pac J Clin Nutr.

[16] Franks PW, Ravussin E, Hanson RL, Harper IT, Allison DB, Knowler WC, Tataranni PA, Salbe AD (2005). Habitual physical activity in children: the role of genes and the environment. Am J Clin Nutr.

[17] Stubbe JH, Boomsma DI, Vink JM, Cornes BK, Martin NG, Skytthe A, Kyvik KO, Rose RJ, Kujala UM, Kaprio J (2006). Genetic influences on exercise participation in 37,051 twin pairs from seven countries. PLoS One.

[18] Fernandes MM, Sturm R (2011). The role of school physical activity programs in child body mass trajectory. J Phys Act Health.

[19] Halliday TJ, Kwak S (2009). Weight gain in adolescents and their peers. Econ Hum Biol.

[20] O'Connor DP, Lee RE, Mehta P, Thompson D, Bhargava A, Carlson C, Kao D, Layne CS, Ledoux T, O'Connor T (2015). Childhood obesity research demonstration project: cross-site evaluation methods. Child Obes.

[21] Centers for Disease Control and Prevention: National Health and Nutrition Examination Survey: Anthropometry Procedures Manual [https://www.cdc.gov/nchs/data/nhanes/nhanes_07_08/manual_an.pdf] Accessed 12 Dec 2017.

[22] Centers for Disease Control and Prevention: A SAS Program for the CDC Growth Charts [https://www.cdc.gov/nccdphp/dnpao/growthcharts/resources/sas.htm] Accessed 12 Dec 2017.

[23] Foltz JL, Belay B, Dooyema CA, Williams N, Blanck HM (2015). Childhood obesity research demonstration (CORD): the cross-site overview and opportunities for interventions addressing obesity community-wide. Child Obes.

[24] Flegal KM, Wei R, Ogden CL, Freedman DS, Johnson CL, Curtin LR (2009). Characterizing extreme values of body mass index-for-age by using the 2000 Centers for Disease Control and Prevention growth charts. Am J Clin Nutr.

[25] Davison KK, Falbe J, Taveras EM, Gortmaker S, Kulldorff M, Perkins M, Blaine RE, Franckle RL, Ganter C, Baidal JW (2015). Evaluation overview for the Massachusetts childhood obesity research demonstration (MA-CORD) project. Child Obes.

[26] University of Texas School of Public Health: School Physical Activity and Nutrition (SPAN) project - Student Survey [https://sph.uth.edu/dotAsset/b94d672a-ca59-4bcb-b2e9-1d893c503764.pdf, https://sph.uth.edu/dotAsset/4d09697e-e692-4e66-816e-d9d6ccb35499.pdf] Accessed 12 Dec 2017.

[27] National Institute of Health: CATCH Kids Club After-school Student Questionnaire [https://www.nhlbi.nih.gov/health/educational/wecan/downloads/CKC-questionnaire.pdf] Accessed 12 Dec 2017.

[28] Centers for Disease Control and Prevention: Youth Risk Behavior Survey (YRBS) [http://www.cdc.gov/healthyyouth/yrbs/pdf/questionnaire/2009MiddleSchool.txt] Accessed 12 Dec 2017.

[29] Ihmels MA, Welk GJ, Eisenmann JC, Nusser SM, Myers EF (2009). Prediction of BMI change in young children with the family nutrition and physical activity (FNPA) screening tool. Ann Behav Med.

[30] Fogelholm M, Nuutinen O, Pasanen M, Myohanen E, Saatela T (1999). Parent-child relationship of physical activity patterns and obesity. Int J Obes Relat Metab Disord.

[31] Williams SL, Mummery WK (2011). Links between adolescent physical activity, body mass index, and adolescent and parent characteristics. Health Educ Behav.

[32] Hoelscher DM, Day RS, Kelder SH, Ward JL (2003). Reproducibility and validity of the secondary level school-based nutrition monitoring student questionnaire. J Am Diet Assoc.

[33] Pei Z, Flexeder C, Fuertes E, Standl M, Berdel D, von Berg A, Koletzko S, Schaaf B, Heinrich J (2014). Mother's body mass index and food intake in school-aged children: results of the GINIplus and the LISAplus studies. Eur J Clin Nutr.

[34] Trost SG, Pate RR, Sallis JF, Freedson PS, Taylor WC, Dowda M, Sirard J (2002). Age and gender differences in objectively measured physical activity in youth. Med Sci Sports Exerc.

[35] Fakhouri TH, Hughes JP, Brody DJ, Kit BK, Ogden CL (2013). Physical activity and screen-time viewing among elementary school-aged children in the United States from 2009 to 2010. JAMA Pediatr.

[36] Dumith SC, Gigante DP, Domingues MR, Kohl HW (2011). Physical activity change during adolescence: a systematic review and a pooled analysis. Int J Epidemiol.

[37] Lau EY, Faulkner G, Qian W, Leatherdale ST (2016). Longitudinal associations of parental and peer influences with physical activity during adolescence: findings from the COMPASS study. Health Promot Chronic Dis Prev Can.

[38] Johansson E, Mei H, Xiu L, Svensson V, Xiong Y, Marcus C, Zhang J, Hagstromer M (2016). Physical activity in young children and their parents-an early STOPP Sweden-China comparison study. Sci Rep.

[39] Drenowatz C, Erkelenz N, Wartha O, Brandstetter S, Steinacker JM (2014). Parental characteristics have a larger effect on children's health behaviour than their body weight. Obes Facts.

[40] Field AE, Gillman MW, Rosner B, Rockett HR, Colditz GA (2003). Association between fruit and vegetable intake and change in body mass index among a large sample of children and adolescents in the United States. Int J Obes Relat Metab Disord.

